# Diffuse Alveolar Hemorrhage After Pediatric Hematopoietic Stem Cell Transplantation

**DOI:** 10.3389/fonc.2020.01757

**Published:** 2020-09-09

**Authors:** Kimberly Fan, Jennifer McArthur, R. Ray Morrison, Saad Ghafoor

**Affiliations:** ^1^Division of Pediatric Critical Care, University of Tennessee Health Science Center, Memphis, TN, United States; ^2^Division of Critical Care, St. Jude Children's Research Center, Memphis, TN, United States

**Keywords:** diffuse alveolar hemorrhage, hematopoietic cell transplant (HCT) complications, pediatric oncology, glucocorticoids, thrombotic microangiopathy, graft vs. host disease, pulmonary hemorrhage, extracorporeal membrane oxygenation (ECMO)

## Abstract

Pulmonary complications are common following hematopoietic cell transplantation (HCT) and contribute significantly to its morbidity and mortality. Diffuse alveolar hemorrhage is a devastating non-infectious complication that occurs in up to 5% of patients post-HCT. Historically, it carries a high mortality burden of 60–100%. The etiology remains ill-defined but is thought to be due to lung injury from conditioning regimens, total body irradiation, occult infections, and other comorbidities such as graft vs. host disease, thrombotic microangiopathy, and subsequent cytokine release and inflammation. Clinically, patients present with hypoxemia, dyspnea, and diffuse opacities consistent with an alveolar disease process on chest radiography. Diagnosis is most commonly confirmed with bronchoscopy findings of progressively bloodier bronchoalveolar lavage or the presence of hemosiderin-laden macrophages on microscopy. Treatment with glucocorticoids is common though dosing and duration of therapy remains variable. Other agents, such as aminocaproic acid, tranexamic acid, and activated recombinant factor VIIa have also been tried with mixed results. We present a review of diffuse alveolar hemorrhage with a focus on its pathogenesis and treatment options.

## Introduction

Hematopoietic cell transplant (HCT) is increasingly used as a treatment for various malignant and non-malignant disease processes. Post-transplant, pulmonary complications are common, occurring in up to 40–60% of transplant recipients, and contribute to significant morbidity and mortality (1). Diffuse alveolar hemorrhage (DAH) is a clinical syndrome characterized by dyspnea, pulmonary infiltrates on chest radiography, and progressively bloodier bronchoalveolar lavage on bronchoscopy (2). DAH was first described by Robbins et al. in adults following autologous HCT for various oncologic processes (3, 4). Since then, there have been several case series examining DAH in patients who have received autologous and allogeneic HCT for both malignant and non-malignant diseases (4, 5). The incidence of DAH is typically reported between 2.5 and 5% of patients undergoing allogeneic HCT (4–7); however, incidence has been reported to occur in up to 40% of HCT recipients in some series (1). DAH historically yielded a high overall mortality rate between 64 and 100%, most commonly due to respiratory failure, multiorgan failure, and sepsis (2, 4, 6, 8, 9). While more recent case series describing the use of newer treatment agents, such as activated human recombinant factor VII, have reported improved mortality rates below 50%, (10–13), there remains a great need for improved understanding of this disease process in order to develop precision treatment modalities.

## Pathophysiology

Alveolar hemorrhage results from damage of the pulmonary microcirculation, loss of integrity in the alveolar-capillary basement membrane, and accumulation of red blood cells in the alveolar space (14). It presents with a spectrum of histologic findings, including pulmonary capillaritis, bland pulmonary hemorrhage, and diffuse alveolar damage (DAD) (14). Of these, pulmonary capillaritis is the most commonly described histologic subtype overall and is frequently seen in the setting of systemic vasculitis or connective tissue disorders (14). However, there is significant overlap of DAD and DAH on post-mortem exam in HCT patients, suggesting that not only is DAD the most common histologic subtype of DAH in this patient population, but that DAD likely contributes to the development and progression of DAH (14–16).

**Figure 1:**
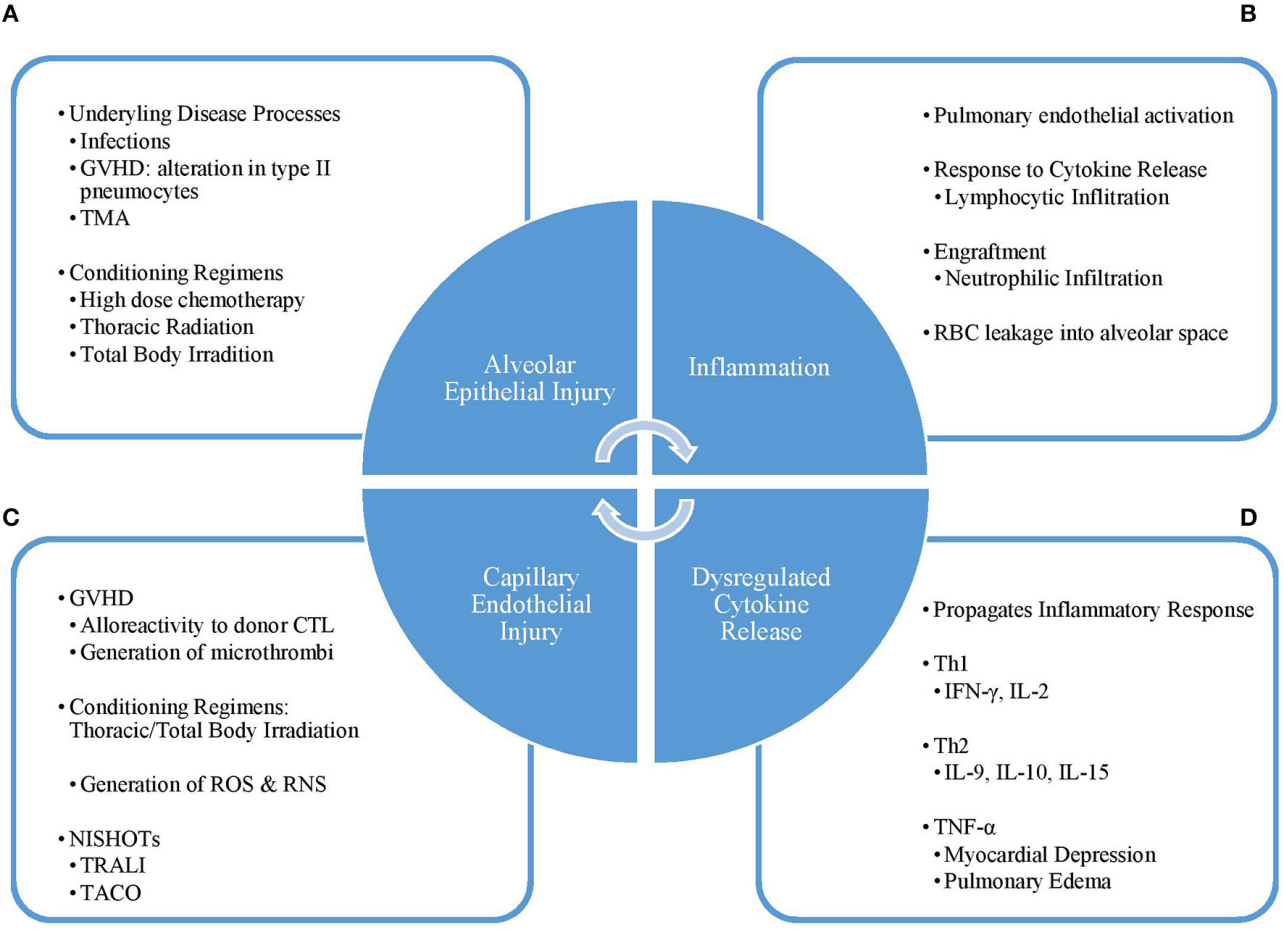
Pathogenesis cycle of DAH. **(A)** The initial injury to the lung alveoli is caused by condition regimens or other disease processes such as infection, TMA, or GVHD leading to **(B)** influx of inflammatory cells and molecules into the alveoli and **(C)** a dysregulated pattern of cytokine release. This response leads to **(D)** damage of the capillary endothelium, which can be worsened by supportive therapies, such as TRALI and TACO. Disruption of the capillary endothelium, and thus the alveolar-capillary complex, furthers lung injury and leads back into a vicious cycle. GVHD, graft vs. host disease; TMA, thrombotic microangiopathy; Th1, type 1 helper T-cell; Th2, type 2 helper T-cell; IFN-γ, interferon-gamma; IL, interleukin; TNF-α, tumor necrosis factor-alpha; CTL, cytotoxic T-lymphocyte; ROS, reactive oxygen species; RNS, reactive nitrogen species; NISHOT, non-infectious serious hazards of transfusions; TRALI, transfusion-related acute lung injury; TACO, transfusion-associated circulatory overload.

The exact pathogenesis of DAH in the post-HCT population has not been well-understood but is thought to result from a direct insult to the lungs followed by significant inflammation and cytokine release leading to damage of the alveolar capillaries (2, 8, 14) (Figure 1).

Direct injury to the lung results from underlying systemic diseases such as occult infections, graft vs. host disease (GVHD), thrombotic microangiopathy, and the use of conditioning regimens including high-dose chemotherapy agents, thoracic irradiation, and total body irradiation (2, 7, 8, 17). The alveolar-capillary complex is especially sensitive to damage resulting from thoracic and total body irradiation, which causes injury through generation of reactive oxygen and nitrogen species that results in damage to the alveolar epithelium, loss of endothelial integrity of the pulmonary capillaries leading to loss of barrier function, and reduction of lung perfusion promoting the development of hypoxia (18, 19).

Diffuse alveolar damage is a common finding in both DAH and idiopathic pneumonia syndrome (IPS), suggesting that DAH could possibly be a distinct subset of IPS which involves alveolar injury resulting from exposure to intensive conditioning regimens, radiation therapy, and occult infections (2, 20). While IPS and DAH share many similar clinical features, DAH is distinguished from IPS in that it maintains a pro-inflammatory cytokine environment with very little fibrotic effect (2). On the other hand in IPS, as leukocyte activation progresses, there is dysregulated wound healing and a shift in the cytokine environment to one that promotes pulmonary fibrosis (21). This was demonstrated by a small study conducted by Vusse et al. in which the authors showed that patients with DAH in the context of IPS had different cytokine concentrations on BAL when compared to that of patients with IPS but no DAH. However, there was no significant differences in serum cytokine concentrations between the two groups suggesting that there is a local pathobiological process (22). While the evidence is limited, this study suggests that intrapulmonary cytokine levels may be a helpful biomarker in diagnosis and development of precise immunomodulatory therapies for DAH.

Exposure to these factors leads to activation of the endothelium in the pulmonary microvasculature and release of various inflammatory mediators. Dysregulated production of cytokines including both Th1 (IFN-γ, IL-2) and Th2 (IL-9, IL-10, IL-15) cytokines as well as an increase in IL-6, IFN-γ-inducible protein 10 and TNF-α (23). Lymphocytic and neutrophilic infiltration into the lungs from cytokine action and engraftment/bone marrow recovery, respectively, further potentiates pulmonary tissue damage (3, 21, 24). Neutrophils and neutrophilic products can be found in the lower respiratory tract even in the setting of peripheral neutropenia (24). Damage to the alveolar-capillary complex allows for leakages of red blood cells into the alveolar space and further potentiates the inflammatory response. (2, 8, 25). In the setting of acute GVHD, alloreactivity of the donor cytotoxic T-cells contribute to endothelial damage in the lungs (17, 25). In human and murine models, there is an increase in neutrophils and microthrombi in the alveolar capillaries, and alterations in the type 2 pneumocytes are also seen (26).

Aside from the direct pulmonary effects, the release of cytokines such as TNF-α can lead to myocardial depression (27). The inevitable transfusion of blood products to correct coagulopathy/thrombocytopenia in the peri-HCT period can result in transfusion-related circulatory overload (TACO) (28). The combination of fluid overload and depressed myocardial function worsen leakage of blood and fluid through the already damaged alveolar capillary endothelium (29).

## Risk Factors

Prior to its description as a complication following HSCT, alveolar hemorrhage was described in various systemic disease processes associated with direct injury or secondary to indirect inflammatory damage to the pulmonary vasculature. Among these were systemic vasculitides, collagen vascular diseases, mitral valve disease, infections, and medications such as penicillamine, abciximab, nitrofurantoin, propylthiouracil, and amiodarone (2, 8, 30). Since then, several factors have been implicated in the development of DAH post-HCT. Initial reports of DAH were described in patients >40 years old who underwent autologous HCT for an underlying solid malignancy. The onset of symptoms was at time the time of engraftment and associated with fever and severe mucositis (3). Intensive pre-transplant conditioning with total body irradiation or thoracic radiation, severe graft-vs.-host disease (GVHD), second transplant, dimethyl sulfoxide used as a cryopreservative, and acquisition of neutrophil, T cell, and B cell immunologic deficits secondary to the underlying disease process or primary treatment have subsequently been identified as risk factors for the development of alveolar hemorrhage (7, 16, 17, 20, 31–33). Additionally, the use of certain medications such as sirolimus and defibrotide to treat GVHD and sinusoid obstruction syndrome, respectively, have also been associated with the development of pulmonary/diffuse alveolar hemorrhage post-HCT (34, 35). The overall incidence of DAH is similar between autologous and allogeneic HCT (2) with reported incidence of 0.9–21% and 2.3–12.2% following autologous (3, 6, 36) and allogeneic (4–6, 32, 33, 37) transplantation, respectively. Use of umbilical cord blood graft was associated with a higher incidence of DAH compared to use of peripheral blood or bone marrow grafts (32, 37). In patients transplanted with peripheral blood or bone marrow grafts, delayed neutrophil engraftment and graft failure were identified as risk factors for development of DAH (32).

Renal insufficiency is associated with the development of DAH. The association was thought to be secondary to uremia-induced platelet dysfunction or fluid overload (3). However, as more is understood about transplant associated thrombotic microangiopathy (TMA), it is thought TMA is more likely the link between DAH and renal failure.

In pediatrics, there is a trend toward increased DAH in patients who received allogeneic HCT for non-malignant diseases rather than malignancies (4). In these patients, age <1 year, early WBC recovery and fever are associated with the development of DAH (4, 37). The role of thrombocytopenia in the development of DAH is unclear. Most patients are thrombocytopenic at the onset of DAH and prolonged thrombocytopenia may be associated with development of DAH (4, 5, 32). However, neither the platelet nadir nor platelet transfusions appears to affect disease outcome (3, 4, 17, 38). As thrombocytopenia is a component of thrombotic microangiopathy (TMA), which can lead to DAH, it could be the link in certain cases. Coagulopathy in uncommon and does not appear to play a significant role in DAH (32, 33, 39).

## Clinical Presentation and Diagnosis

While the onset of DAH is typically within the first 30 days post-HCT, it has been reported after the first month of transplant (2, 4, 8, 40). There are no standardized clinical, radiographic, or laboratory markers to make the diagnosis. Due to the lack of standardization, there is significant heterogeneity in diagnostic criteria. Historically, various clinical criteria have been used to diagnose alveolar hemorrhage, including a high percentage (≥ 20%) of hemosiderin-laden alveolar macrophages, Golde score >100, indicating a high hemosiderin content within macrophages, and progressively bloodier BAL fluid on bronchoscopy (8, 41). It is now classically characterized as a constellation of findings including dyspnea, hypoxemia, diffuse pulmonary infiltrates on chest radiography, and progressively bloodier bronchoalveolar lavage return on bronchoscopy (2, 4, 5, 8, 14, 31). There should be evidence of widespread alveolar injury and abnormal pulmonary physiology associated with an increased alveolar to arterial oxygen gradient and restrictive ventilatory defect (2). While patients often present with fever, cough, and dyspnea, hemoptysis is surprisingly uncommon in adults (5, 8, 20) but is more commonly reported in children (4). Abnormalities in chest radiography can be seen prior to the clinical symptoms of DAH (36). Radiographic findings are initially non-specific and often characterized by diffuse pulmonary infiltrates, most pronounced in the bilateral perihilar areas and lower lobes (4, 20, 36, 42). As the disease progresses, worsening of the chest radiograph to a diffuse, severe reticular alveolar pattern is appreciated (36). Findings on computed tomography are similar to those seen on plain radiography, with predominately ground glass opacities and/or a reticular pattern of injury, consistent with acute alveolar/interstitial disease (42). Oftentimes, respiratory compromise or a new infiltrate on chest radiography leads to bronchoscopy (6), which is used in these patients to establish the diagnosis and exclude other causes for these symptoms such as infection or recurrence of malignancy. During bronchoscopy, there should be progressively bloodier fluid returned or hemosiderin-laden macrophages on bronchoalveolar lavage samples with sequential instillation of saline (4, 14, 43). However, an early study called into question the utility of BAL in the diagnosis of DAH (15), demonstrating that 7/13 patients with hemorrhagic BAL fluid did not have histologic evidence of DAH on post-mortem exam while 4/8 with DAH did not have hemorrhagic BAL fluid. Agusti et al. hypothesized that these findings may be explained by processes that cause hemorrhagic exudates in the alveolar spaces such as CMV pneumonitis or pulmonary aspergillosis and that bronchoscopy may not have explored the area of DAH, respectively (15). While pulmonary infections can lead to alveolar hemorrhage, the diagnosis of diffuse alveolar hemorrhage in the post-HCT period is reserved for alveolar hemorrhage from a non-infectious etiology (2, 8). As such, many studies exclude those with signs of infection such as with a recent positive bronchoalveolar lavage culture or localized pulmonary hemorrhage from other causes such as chronic bronchitis, bronchiectasis, or tumors (2, 5, 8, 31).

## Outcomes

Outcomes of DAH following HCT have historically been dismal, with most with in-hospital mortality commonly cited between 64 and 100% (3, 4, 9, 36, 44, 45). Most, if not all, patients require intensive care and mechanical ventilatory support (4–8, 31, 32, 43, 45, 46). Poor prognostic factors include allogeneic transplant, umbilical cord blood as the graft source, infection-related alveolar hemorrhage, and need for mechanical ventilation (1, 8, 32). Factors associated with improved outcomes include early onset (<30 days after HCT) of DAH, which is also associated with an improved response to systemic glucocorticoids, and DAH occurring during the periengraftment period (+/– 5 days from neutrophil engraftment) (8, 31). The improvement in survival periengraftment is hypothesized to be secondary to the transient nature of engraftment and potential for improvement following the resolution of the periengraftment cytokine storm (31).

## Management

### Supportive Measures

The approach to management of DAH should reflect this entity's diverse and complex pathogenesis. Most patients with DAH require care in an intensive care unit and invasive mechanical ventilation (8). Mechanical ventilation strategies include maintaining a high positive end-expiratory pressure (PEEP) in effort to tamponade capillary bleeding and on occasion escalation to high frequency oscillatory ventilation (HFOV) (13, 39). Supportive measures such as optimization of fluid and electrolyte balance, correction of coagulopathies and prophylactic antibiotics are a mainstay of treatment for DAH (6).

### Transfusion of Blood Products

Most patients are thrombocytopenic at time of DAH onset. However, outcomes have not been shown to be affected by either the nadir platelet count or its correction by platelet transfusions (3, 4, 38). Coagulopathy associated with abnormalities in prothrombin time (PT), international normalized ratio (INR), and activated partial thromboplastin time (aPTT) is uncommon in patients with DAH (32, 33, 39). While transfusion of blood products, particularly platelets, is common in the management of DAH, the unrestrained use of platelet transfusions is not without risks. Platelet and red blood cell (RBC) transfusions are independently associated with a higher risk of venous thromboembolisms, arterial thromboembolisms, and in-hospital mortality in hospitalized oncology patients (47). Platelet transfusions have been implicated in transfusion reactions such as allergic reactions, febrile non-hemolytic reactions, transfusion-associated sepsis, and transfusion-related acute lung injury (TRALI) (48). The potential for injury is further elevated in these patients as HCT has been identified as risk factor for developing TRALI, and; during periods of inflammation, such as is seen in DAH, platelet transfusions may increase vascular permeability and thereby worsening capillary leak (48–50).

As traditional transfusion practices have not shown to impact outcomes in DAH, thromboelastography (TEG) and rotational thromboelastometry (ROTEM) TEG/ROTEM-guided transfusion may be considered and is an area for future research. They have been used to assess global clotting function in various bleeding disorders, during cardiac surgery, liver transplantation, and traumas (51). In patients with hemophilia, TEG/ROTEM assay results correlated well with phenotypic disease severity. They are useful for monitoring response to bypassing agents, such as recombinant activated FVII (51). Furthermore, a Cochrane Review of TEG/ROTEM use in cardiac surgery showed a trend toward improved mortality and an overall significant reduction in pooled red blood cell, fresh frozen plasma, and platelet requirements using TEG/ROTEM-guided transfusions (52).

Anemia is also a common finding in the critically-ill pediatric population, occurring in 74% of these patients with 49% receiving one or more RBC transfusions during their PICU course (53). While RBC transfusions can correct severe anemia, thus increasing the oxygen content of blood, it does not necessarily increase tissue oxygen delivery and oxygen consumption (54). Furthermore, they have been associated with non-infectious serious hazards of transfusions (NISHOTs) such as TRALI/TACO, and mortality (53–55). In 2007, Lacroix et al. showed that in hemodynamically stable patients, a restrictive strategy of RBC transfusions, defined as a hemoglobin threshold of 7 gram/deciliter, did not result in an increase in new or progressive multiple-organ dysfunction syndrome and that patients received 44% fewer transfusions than those in the liberal-transfusion, defined as a hemoglobin threshold of 9.5 gram/deciliter, group (56). In 2018, the Pediatric Critical Care Transfusion and Anemia expertise Initiative (TAXI) published good practice guidelines and recommendations for the use of RBC transfusions. The guidelines supported Lacroix's restrictive transfusion threshold of 7 g/dl in hemodynamically stable patients and urged clinicians to regard the need for transfusion in the context of the patient's broader clinical picture rather than simply looking at a threshold number (54, 56, 57).

### Glucocorticoids

Glucocorticoids (GC) have been used for many years in various inflammatory and autoimmune disorders. Cases of DAH which coincide with marrow recovery are thought to be due to an inflammatory diffuse alveolar damage potentially responsive to glucocorticoids. This has made glucocorticoids the mainstay of treatment (2, 4, 8, 24). First demonstrated by Metcalf et al. patients treated with high dose steroids, defined as a daily dose of methylprednisolone 30 mg or equivalent, had an increase in overall survival and decreased need for invasive mechanical ventilation without an increase in rates of systemic bacterial or fungal infections when compared to patients treated with low-dose steroids or supportive care alone (24).

The mechanism by which glucocorticoids modulate inflammation occurs through genomic and non-genomic pathways (Table 1). Within the cytoplasm, glucocorticoid receptors (GCR) bind to free glucocorticoids with high affinity (58). The glucocorticoid-glucocorticoid receptor complex enters the nucleus and binds to specific DNA sequences known as either positive or negative glucocorticoid-responsive elements (GREs). When bound to positive GREs, the glucocorticoid-GCR complexes exerts a direct genomic effect through activating the transcription of anti-inflammatory proteins such as IL-10, Annexin-1, mitogen-activated protein kinase (MAPK) phosphatase-1, and inhibitors of nuclear factor NF-kB (59, 60). Annexin-1 blocks the release of arachidonic acid and its conversion to various eicosanoids such as prostaglandins, thromboxanes, prostacyclins, and leukotrienes (58). The glucocorticoid-GCR complex induces indirect genomic effects by competitive binding with (NF)-kB and activator protein 1 (AP-1) thereby suppressing the expression of pro-inflammatory molecules (COX-2, cytokines such as IL-2, TNF-α, IFN-γ, chemokines, cell adhesion molecules, complement factors, and their receptors) (58–62). It is thought that most of the desired anti-inflammatory effects are secondary to the suppressive effects on gene transcription (60). Another genomic effect by which glucocorticoids inhibit inflammation is through decreasing mRNA stability for inflammatory proteins such as vascular endothelial growth factor and cyclooxygenase 2 (63). The genomic effects of glucocorticoids can be appreciated at lower doses but require a longer onset of action, at least 30 min but sometimes taking up to hours or days, than the non-genomic effects (58, 60, 63, 64). The non-genomic effects occur quickly, within seconds to minutes following administration of a glucocorticoid (58, 63, 65).

**Table 1:** Comparison between genomic and non-genomic effects of glucocorticoids.

	**Genomic**	**Non-genomic**
Dose	Low dose resulting in saturation of the cytoplasmic GC receptors	>100 mg, up to 1 g
Duration of onset	>30 min	Seconds to minutes
Actions	Direct: forms GC/GCR complexes in cytoplasm which enter the nucleus and binds to/regulates transcription of GRE's Indirect: suppress expression of pro-inflammatory molecules through competitive binding/protein-protein interactions	Reduce intracellular free calcium through inhibition of calcium/sodium cycling, thereby inhibiting neutrophil degranulation Increase proton leak and inhibit oxidative phosphorylation in mitochondria thereby decreasing ATP production Promote integrity of alveolar-capillary complex through activation of eNOS, decrease exudative leakage into the airways

*GC, glucocorticoid; GCR, glucocorticoid receptor; GRE, glucocorticoid-responsive elements, ATP, adenosine triphosphate, eNOS, endothelial nitric oxide synthase*.

Glucocorticoids inhibit the cycling of sodium and calcium across the plasma membrane, reducing the availability of intracellular free calcium, which inhibits neutrophil degranulation (58, 60, 66). Within the mitochondria, they increase proton leak and inhibit oxidative phosphorylation, decreasing production of ATP, an essential energy source of cytokine synthesis and antigen processing/presentation by macrophages (60). Additionally, glucocorticoids contribute to the integrity of the alveolar-capillary complex through activation of the phosphatidylinositol-3-hydroxykinase (PI3K/Akt) signaling pathway which leads to the production of nitric oxide (NO), a mediator of vascular integrity with anti-inflammatory properties (58, 67, 68). Glucocorticoids promote vasoconstriction of the airway vasculature via alpha adrenergic effects and by potentiating angiotensin II (58) and inhibits platelet activating factor and in turn decreases exudative leakage in the airways (69, 70).

Glucocorticoid dosing varies widely and can range up to a 200-fold difference depending on the indication for use (64, 71, 72). The genomic effects of glucocorticoids can be elicited with usage of relatively lower doses, as low as 7.5–100 mg prednisone equivalent a day used in maintenance therapy for rheumatologic conditions saturating <50% of the glucocorticoid receptors (64). However, in order for them to have maximal genomic and non-genomic anti-inflammatory effects, very high doses, defined as over 250 mg prednisone equivalent a day, are required (64). Even higher doses of up to 1,000–2,000 mg of methylprednisolone equivalent per day, referred to as pulse dosing, are used in treatment of acute rheumatologic disease exacerbations in adults (73). This dosing in adults translates to ~15–30 mg/kg/day methylprednisolone equivalents in children (73). While methylprednisolone is most often used for pulse-dosing and has the advantage of faster penetration into the cellular membrane and thus onset of action, dexamethasone, another corticosteroid with high glucocorticoid effects, has been used in a pulse-dose manner (73, 74). When administered at 4–5 mg/kg up to 20 mg/dose, dexamethasone is a more potent anti-inflammatory agent due to its increased affinity for glucocorticoid receptors and non-genomic effects when compared to methylprednisolone (73–75). At these doses, not only is there 100% saturation of the cytosolic glucocorticoid receptors, thus exerting full genomic effects, there is also full elicitation of the more immediate, non-genomic effects (64). It is likely that the addition of the non-genomic effects of glucocorticoids contribute significantly to the termination of acute exacerbations in these inflammatory processes (64).

Many adverse effects of glucocorticoid therapy have been described including hemodynamic changes (hypertension, bradycardia) associated with intravenous infusions, loss of bone mass, suppression of the HPA axis, weight gain, hyperglycemia/diabetes mellitus, cardiovascular disease, myopathy, cataracts, psychiatric disturbances, growth suppression in children, and increased infection risk (73, 76). The dosing over which these adverse effects develop have not been defined but appear to be related to both average and cumulative dose (76, 77). The rationale for pulse dosing is to maximize the immediate non-genomic effects leading to faster recovery of clinical symptoms, minimize the inflammatory damage from disease, and limit the adverse effects associated with long term glucocorticoid use (73). Treatment at these doses are limited in duration of therapy and require either discontinuation or rapid decreases after a maximum of 5 days (64, 73, 76).

Glucocorticoid dosing in the treatment of adult DAH patients has not been standardized (Table 2). While the initial report by Metcalf et al. supported the use of over 30 mg of MP equivalent per day, there is a paucity of studies comparing different treatment doses. Rathi et al. compared low, medium, and high dose, defined as <250, 250–1,000, and >1,000 mg/day of MP, respectively, and found a significantly lower ICU and hospital mortality in patients treated with lower dose steroids compared to those treated with medium or high doses (45). However, this study was limited by its retrospective nature. Furthermore, while there was no significant difference in mortality predictive indices between the treatment groups at time of admission, the sicker patients at time of DAH diagnosis may have been placed on higher doses of steroids, thereby confounding the results (45). In other case series and reports, high doses of glucocorticoids have been used- methylprednisolone 30 mg/kg/day to a max of 2 gram day is used for 3–5 days which is followed by a slow taper over 2–4 weeks (2, 6, 8).

**Table 2:** Glucocorticoid use in adult patients with DAH.

**Author/year**	**Number of transplants**	**Cases of DAH, N (%)**	**Age in years, median (range)**	**HCT reason, N**	**Type/source, N**	**DAH onset after HCT, median (range), in days**	**Infectious agent^a^, N**	**Glucocorticoid regimen, (N)**	**Adjunctive directed therapies, (N)**	**Mortality, N (%)**	**Cause of death, (N)**	**Complications of therapies**
**Adults**												
Afessa et al. (8)	1,215	48 (4)	Mean: 47.7 SD: 12	Malignancy (hematologic): 42 Malignancy (non-hematologic): 1 Non-malignant: 5	Allo: 23 Auto: 25 BM: 16 PBSC: 32	23.5 (4–1,330)	None ^b^	Initial dose (44): IV MP 60 mg – 2 g/day X 1-14 days, median 3d Total treatment duration 2–117 days, median 22d	None	Hosp: 23 (48)	RF (15) Sepsis (4) MOF (1) Other ^c^ (3)	None noted
Chao et al. (7)	77	4 (5.2)	(28–50)	Malignancy (hematologic): 4 Malignancy (non-hematologic): 0 Non-malignant: 0	Allo: 0 Auto: 4 Source UNS	(14–20)	None	Initial dose (4): IV MP 1 gram/day X 3 days Dose reduced by 50% every 3 days until 60 mg daily dose; then, transitioned to a prednisone taper over 2 months	None	Hosp: 0 (0)	N/A	No infectious
Gupta et al. (1)	UNS	87	48 (22–76)	Malignancy (hematologic): 79 Malignancy (non-hematologic): 1 Non-malignant: 7	Allo: 74 Auto: 13 BM: 29 PBSC: 54 UCB: 2	3 (0–105)	+BAL, total: 34 Fungal: 18 Bacterial: 6 Viral: 7 Parasitic: 3	Initial dose (87): IV MP ≥ 250 mg/day (or equivalent), unspecified duration of treatment	rFVIIa (24) Desmopressin (4) ACA (3)	6-month: (62.2)	RF (28) MOF (14) Other ^d^ (16)	None noted
Keklik et al. (32)	1,228	59 (4.8)	32	Malignancy (unspecified): 50 Non-malignant: 9	Allo: 59 Auto: 0 BM: 9 PBSC: 10 UCB: 40	30 (3–168)	Systemic infection: 27% +BAL: 19%	Initial dose (52):-Adults: IV MP 1 gram/day, divided BID, X 3 days-Pediatrics: 500 mg/m2/day, divided BID, X 3 daysDose reduced by 50% every 3 days until 60 mg daily dose; then, tapered over 2 months. Pediatrics similar taper to adults	Anti-TNF (7) ^e^ -Etanercept (6) -Infliximab (1)	60-day: (55.8)	UNS	None noted
Lewis et al. (6)	922	23 (2.5)	36 (24-62)	Malignancy (hematologic): 23	Allo: 19 Auto: 4 BM: 18 PBSC: 5	19 (5–34)	None	Initial dose (15): IV MP 250 mg - 2 g/day, unspecified duration of treatment	None	60 day: 17 (73.9)	RF (3) Sepsis (3) MOF (11)	None noted
Majhail et al. (31)	1,919	116 (6)	40 (0.6–71)	Malignancy (hematologic): 100 Non-malignant: 16	Allo: 103 Auto: 13 BM: 48 PBSC: 42 UCB: 26	28 (3–785)	Total: 71 +BAL: 50 +BCX: 12 +BAL& BCX: 9	Initial dose (96): IV MP 1 gram/day X 3 days Dose reduced by 50% every 3 days until 60 mg daily dose; then, tapered over 2 months	None	60-day: 86 (74.1)	UNS	None noted
Metcalf et al. (24)	603	65 ^f^	Adults > 18 years	Malignancy (hematologic): 38 Malignancy (non-hematologic): 24 Non-malignant: 3	Allo: 8 Auto: 57 BM: 65	UNS	None	Low dose (10): < 30 mg MP equivalent/day High dose (43): > 30 mg MP equivalent/day Typical initial high dose: IV MP 500-1,000 mg/day (divided q6h) X 4–5 days, tapered over 2–4 weeks	None	Hosp:-No GC: 11(91.7)-Low dose: 9 (90)-High dose: 29 (67.4)	UNS for 60 day mortality	High dose without increase in development of infections
Raptis et al. (33)	74	4 (5.4)	(23–43)	Malignancy (hematologic): 4	Allo: 4 Auto: 0 BM: 2 PBSC: 2	(0–23)	None	Initial dose (4): IV MP 500 mg – 2,000 gday X 1–5 days Subsequently tapered, unspecified duration of treatment	None	60 day: 2 (50)	RF&MOF (2)	CMV + on repeat BAL (1)
Rathi et al. (45)	2,650	119 (4.5)	Adults > 18 years	Malignancy (hematologic): 117 Malignancy (non-hematologic): 2	Allo: 100 Auto: 19 BM: 26 PBSC: 72 UCB: 20	UNS for overall cohort	+Resp culture: 35%	Low dose (37): IV MP < 250 mg/day Medium dose (51): IV MP 250–1,000 mg/day High dose (31): IV MP ≥ 1,000 mg/day All dosing with subsequent taper	ACA (82)	ICU: (51) Hosp: (76) 60-day: (78)	UNS	None noted
Wanko et al. (43)	UNS	15^g^	41 (20–54)	Malignancy (hematologic): 13 Malignancy (non-hematologic): 1	Allo: 14 Auto: 0 Source UNS	40.5 (11–177)	Total: 6 Yeast: 4 HSV: 1 Resp Viral: 2^h^	Initial dose (15): IV MP 1 gram/day, divided q6h Dose decreased by 50% every 3 days	ACA (9)	100-day: (60)	RF (8) UNS (1)	None noted

*DAH, diffuse alveolar hemorrhage; GC, glucocorticoid; Allo, allogeneic; Auto, autologous; BM, bone marrow; PBSC, peripheral blood stem cell; UCB, umbilical cord blood; UNS, unspecified; IV, intravenous; MP, methylprednisolone; Hosp, hospital; BAL, bronchoalveolar lavage; BCX, blood culture; TNF, tumor necrosis factor; RF, respiratory failure; MOF, multiple organ failure; CMV, cytomegalovirus; ACA, aminocaproic acid; HSV, herpes simplex virus*.

a *Identified at time of DAH*;

b *2 patients with evidence of bronchopneumonia on autopsy*;

c *Intracranial bleed (1), Multifocal leukoencephalopathy (1), underlying disease progression (1)*;

d *Disease progression (8), graft vs. host disease (2), unspecified (4)*;

e *3 patients predominately treated with anti-TNF therapy for graft vs. host disease*;

f *63 patients with 65 episodes of diffuse alveolar hemorrhage*;

g *14 patients with 15 episodes of diffuse alveolar hemorrhage*;

h *1 patient was also positive for yeast*.

Reports of glucocorticoid use of DAH following HCT in the pediatric population are limited to small, retrospective reports (Table 3). Ben-Abraham et al. reported a series of 6 children who were treated with moderate glucocorticoid doses (6 mg/kg methylprednisolone) in which only 1 survived the initial injury (4). Heggen et al. reported better overall survival (4/7) but no significant difference between patients treated with 1,000 mg/day (3/4) and <500 mg/day (1/3) of methylprednisolone (5). While one cannot draw definite conclusions between these two studies, it appears that there is a trend toward improved survival with use of higher glucocorticoid doses. Prospective studies are greatly needed to develop treatment regimens as mortality for DAH remains high. At this time, in cases of DAH thought to be caused by inflammation-induced alveolar damage, we advocate for the use of high dose glucocorticoid therapy to achieve maximal genomic and non-genomic effects and minimize side effects. In circumstances where underlying infection is suspected, we recommend weaning over 4–8 weeks.

**Table 3:** Glucocorticoid use in pediatric patients with DAH.

**Author/Year**	**Number of transplants**	**Cases of DAH, N (%)**	**Age in years, median (range)**	**HCT reason, N**	**Type/source, N**	**DAH onset after HCT, median (range), in days**	**Infectious agent, N**	**Glucocorticoid regimen, (N)**	**Adjunctive directed therapies, (N)**	**Mortality, N (%)**	**Cause of death, (N)**	**Complications of therapies**
**Pediatrics**												
Ben- Abraham et al. (4)	138	6 (4.3)	(0.17–10)	Malignancy (hematologic): 1 Non-malignant: 5	Allo: 6 Auto: 0 BM: 6	(1–37)	None	Initial dose (6): IV MP 6 mg/kg/day X 3 days	None	Hosp: 5 (83.3)	RF (4) MOF (1)	None noted
Heggen et al. (5)	138	7 (5.1)	13.4 (1.4–15.2)	Malignancy (hematologic): 5 Non-malignant: 2	Allo: 7 Auto: 0 Source UNS	24 (10–50)	None	High dose (4): IV MP 1 gram/day X 3 days Low dose (3): IV MP < 500 mg daily All dosing tapered over 2 months	None	Hosp: 3 (42.9)	RF & MOF (3)	Post-mortem lung culture + yeast and Klebsiella pneumoniae (1) Repeat BAL + Candida albicans (1)

*DAH, diffuse alveolar hemorrhage; HCT, hematopoietic cell transplant; Allo, allogeneic; Auto, autologous; BM, bone marrow; UNS, unspecified; IV, intravenous; MP, methylprednisolone; Hosp, hospital; BAL, bronchoalveolar lavage; RF, respiratory failure; MOF, multiple organ failure*.

### Aminocaproic Acid

Aminocaproic acid (ACA) is an antifibrinolytic agent that has been used with mixed results in the treatment of DAH following HSCT (43, 45). In the original study conducted by Wanko et al. in 2006, there was a significant decrease in 100 day DAH mortality from 83 to 44% in those patients who were treated with a combination of methylprednisolone (250 mg q6h followed by taper of 50% every 3 days) and aminocaproic acid (1,000 mg IV q6h) compared to those who were treated with methylprednisolone alone. There was no major clinically apparent side effect from the addition of aminocaproic acid. Notably, 6/14 patients in this series had an infectious organism identified with bronchoscopy. The authors acknowledge that classically, DAH occurs in the absence of infectious agents, though question the clinical significance of detection of these agents on the development and progression of DAH (43). However, a follow-up study performed by Rathi et al. in 2015, found no significant difference in mortality (30, 60, 100 day, ICU, hospital), number of ventilator days, ICU length of stay, or hospital length of stay between groups who received a combination of steroid and aminocaproic acid (4 gram IV bolus followed by 1 gram/h infusion) or steroids alone. Again, there was no increase in side effects attributed to aminocaproic acid in the cohort of patients who received combination therapy. The authors argue that the discrepancy in efficacy of ACA may be due to a higher severity of illness in this study as compared to the previous one (69% requiring ICU care as opposed to 28% classified as critically ill) (45).

### Nebulized Tranexamic Acid

Tranexamic acid (TXA), also a antifibrinolytic agent acts by binding to plasminogen and inhibits its binding to fibrin and activation to plasmin (78). It has been used in the treatment or prevention of bleeding in hemophilia, ITP, and in operative procedures (79). In patients requiring systemic anticoagulation undergoing dental procedures, the local application of TXA through use of a mouthwash resulted in significantly less post-procedural bleeding without elevated levels of TXA in the serum (80). In a case series reported by Solomonov et al. of six patients with pulmonary hemorrhage of varying etiology treated with direct delivery of TXA either through direct instillation during bronchoscopy or nebulized showed cessation of bleeding in all patients (79). In a cohort of pediatric patients who were diagnosed with DAH, Nebulized TXA alone led to complete or near cessation of bleeding in 10/18 and the addition of nebulized rFVIIa led to hemostasis in an additional 6 patients (12). In this series, a documented respiratory infection was negatively associated with response to nebulized TXA, suggesting that nebulized TXA may not target pulmonary hemorrhage secondary infectious processes (12). O'Neil et al. recently published a case series of 19 pediatric patients with pulmonary hemorrhage who were treated with inhaled or endotracheally instilled TXA in which 18/19 patients had cessation of bleeding (81). No adverse effects related to nebulized TXAs were reported in these series (12, 79, 81).

### Recombinant Activated Factor VII

Intravenous (IV) recombinant activated factor VII (rFVIIa) was initially developed for use in patients with hemophilia A/B with presence of an inhibitor. Under these conditions, an increase in concentration of activated factor VII (FVIIa) produced an increase in the rate of thrombin generation, suggesting that FVIIa is able to bypass the intrinsic coagulation pathway to directly generate thrombin (82, 83). It has since been used in various situations in the pediatric population outside of hemophilia, such as congenital factor VII deficiency, hepatic dysfunction, post-operative bleeding following cardiac surgery, qualitative platelet disorders, and traumatic hemorrhage (84). In recent years, rFVIIa has been used in the treatment of DAH, both in adults (Table 4) and pediatrics (Table 5). It is hypothesized that there are inhibitors of tissue factor, tissue factor pathway inhibitors (TFPI) in inflamed alveoli. These inhibitors prevent FVIIa-tissue factor formation and factor X activation, making the inflamed lungs more susceptible to bleeding (10). Local administration of rFVIIa overcomes the TFPI and restores thrombin generation (10). Few case reports on the use of intravenous rFVIIa have demonstrated cessation of hemorrhage in DAH; however, it often requires higher and more frequent dosing (86, 87).

**Table 4:** Activated human recombinant factor VII use in adult patients with DAH.

**Author/Year**	**Cases of DAH**	**Cases of DAH post-HCT, N (% total cases)**	**Age range, median (range), in years**	**HCT type/source**	**Infectious agent identified, N**	**rFVIIa regimen**	**Adjunctive therapies, (N)**	**Cessation of bleeding, N (%)**	**Mortality, N (%)**	**Cause of death, (N)**	**Thromboembolic complications**
**Adults**											
Baker et al. (10)	6	2 (33.3)	(23–84)	Allo: 1 Auto: 1 Source UNS	None	Intrapulmonary rFVIIa 30-60 mcg/kg daily X 1-2 dose(s)	All received systemic glucocorticoids ACA (3) Other ^a^ (1)	5 (83.3)	DAH episode: 1 (16.7)	RF (1)	None
Estella et al. (46)	2	0 (0)	(39–46)	N/A	None	Intrapulmonary rFVIIa 50 mcg/kg X 1 dose	None	2 (100)	ICU: 0 (0)	N/A	None
Gupta et al. (1)	87	87 (100)	48 (22–76)	Allo: 74 Auto: 13 BM: 29 PBSC: 54 UCB: 2	Total: +BAL: 34 Fungal: 18 Bacterial: 6 Viral: 7 Parasitic: 3	rFVIIa dosing UNS^b^	All received systemic glucocorticoids Desmopressin (4) ACA (3)	UNS	6-month^c^: (62.2) out of all AH	RF (28) MOF (14) Other^d^ (16)	None
Henke et al. (85)	3	1 (33.3)	(23–53)	Allo: 1 BM: 1	None	rFVIIa (unspecified route) 120 mcg/kg q3h X 3 doses rFVIIa (unspecified route) 90 mcg/kg X 2 days rFVIIa (unspecified route) 120 mcg/kg X 1, 180 mcg/kg 6 h later; 90 mcg/kg for rebleed 5 days later	All received MP Plasmapheresis (2) Other^e^ (1)	3 (100)	DAH episode: 0 (0)	N/A	None
Heslet et al. (38)	6	2 (33.3)	(34–63)	Allo: 2 Source UNS	+BAL: 1 (bacterial) UNS pulmonary infection: 1 Systemic CMV: 1	Intrapulmonary/nebulized rFVIIa 50 mcg/kg X 1–3 dose(s)	Standard care including IV/endotracheal TXA and aprotinin infusion IV rFVIIa (1) IV desmopressin (1) IV MP (1)	6 (100)	Overall: 3 (50)	Sepsis (3)	None
Hicks et al. (86)	1	1 (100)	35	Allo: 1 BM: 1	None	Initial: Intravenous rFVIIa 90 mcg/kg q3h X 8 doses Recurrence: Intravenous rFVIIa 90 mcg/kg IV q6h X 16 doses	IV MP/desmopressin/ACA (1)	1 (100)	DAH episode: 0 (0)	N/A	None
Patores et al. (87)	1	1 (100)	48	Allo: 1 Source UNS	None	Intravenous rFVIIa 90 mcg/kg q2h X 2 doses	Systemic GC (1)	1 (100)	Hosp: 0 (0)	N/A	None
Pathak et al. (11)	23	7 (30.4)	Mean 47 SD 19	Type: UNS BM: 7	Not noted	Intravenous rFVIIa 35-120 mcg/kg q2h (4 doses/day) until hemostasis achieved or inadequate response	All received IV MP Plasmapheresis (15) IVIG (1) Cytotoxic drugs^f^ (13)	22 (95.7)	Overall: 8 (34.9)	Sepsis, MOF, progression of underlying disease	None

*DAH, diffuse alveolar hemorrhage; HCT, hematopoietic stem cell transplant; rFVIIa, activated human recombinant factor VII; allo, allogeneic; auto, autologous; UNS, unspecified; ACA, aminocaproic acid; RF, respiratory failure; BM, bone marrow; PBSC, peripheral blood stem cell; UCB, umbilical cord blood; BAL, bronchoalveolar lavage; CMV, cytomegalovirus; MP, methylprednisolone; MOF, multiple organ failure; TXA, tranexamic acid; ETT, endotracheal tube; IVIG, intravenous immunoglobulin; VZV, varicella zoster virus*;

a *Intravenous immunoglobulin/Cyclophosphamide/Rituximab/Plasmapheresis*;

b *24 cases treated with rFVIIa*;

c *Mortality percent of all 87 cases*;

d *Disease progression (8), graft vs. host disease (2), unspecified (4)*;

e *Mycophenolate mofetil/Cyclophosphamide*;

f *Rituximab or Cyclophosphamide*.

**Table 5:** Activated human recombinant factor VII use in pediatric patients with DAH.

**Author/year**	**Cases of DAH**	**Cases of DAH post-HCT, N (% total cases)**	**Age range, median (range), in years**	**HCT type/source**	**Infectious agent identified, N**	**rFVIIa regimen**	**Adjunctive therapies, (N)**	**Cessation of bleeding, N (%)**	**Mortality, N (%)**	**Cause of death, (N)**	**Thromboembolic complications**
**Pediatrics**											
Bafaqih et al. (12)	18	0	2 (IQR 0.94–4.88)	N/A	Resp infection: 10	Nebulized rFVIIa^a^ <25 kg: 35 mcg/kg >25 kg: 50 mcg/kg Administered q4h for maximum of 3 days	All received nebulized TXA	Total: 16 (88.9) rFVIIa: 6/8 (75)	ICU: 2 (11.1% of total, 25% of rFVIIa)	Sepsis (2)	None
Colin et al. (88)	1	0	17	N/A	Bacteremia: 1	Intrapulmonary rFVIIa 50 mcg/kg X 1 dose	Systemic glucocorticoid (1)	1 (100)	Overall: 0 (0)	N/A	None
Larcombe et al. (89)	1	1	2	HCT UNS	Not noted	Intrapulmonary rFVIIa 50 mcg/kg X 1 dose	None	1 (100)	Hosp: 1 (100)	Recurrent pulmonary hemorrhage/withdrawal of care (1)	Occlusion of ETT with blood clot
Park et al. (13)	6	2	11 (0.83–15)	Allo: 2 PBSC: 1 UC: 1	CMV: 1 VZV pneumonia: 1	Intrapulmonary rFVIIa 43–63 (~50) mcg/kg X 1–2 dose(s)	All received systemic glucocorticoids	6	60-day: 2 (33.3)	RF (1) Sepsis (1)	None

*DAH, diffuse alveolar hemorrhage; HCT, hematopoietic stem cell transplant; rFVIIa, activated human recombinant factor VII; allo, allogeneic; UNS, unspecified; RF, respiratory failure; PBSC, peripheral blood stem cell; UCB, umbilical cord blood; CMV, cytomegalovirus; MP, methylprednisolone; MOF, multiple organ failure; TXA, tranexamic acid; ETT, endotracheal tube; IVIG, intravenous immunoglobulin; VZV, varicella zoster virus*;

a *8 cases treated with rFVIIa*.

In effort to decrease systemic effects of rFVIIa, most notably thromboembolic complications, rFVIIa has been delivered directly to the lungs during bronchoscopy or through nebulization (10, 12, 13, 38, 46, 88, 89). Treatment regimens including rFVIIa and dosing are very variable. It has been used as initial therapy in combination with systemic corticosteroids +/− aminocaproic acid or as rescue therapy in cases that are refractory to treatment with other therapies such as corticosteroids, tranexamic acid, aprotinin, aminocaproic acid, plasmapheresis, or desmopressin (10, 38, 85, 86). Heslet et al. first reported the successful intrapulmonary use of rFVIIa in a case series of six patients who developed DAH and failed to respond to other therapies. They received one to three doses of either intrapulmonary or nebulized rFVIIa with all achieving hemostasis and improvement in hypoxia (38). Baker et al. subsequently reported a series of 6 patients treated with intrapulmonary rFVIIa in which 5 patients achieved hemostasis (10). In a trial using rFVIIa for DAH refractory to treatment with nebulized TXA, 6/8 patients showed clinical response to nebulized rFVIIa (12). Park et al. reported a series of 6 pediatric patients with DAH treated with rFVIIa in conjunction with glucocorticoids in which all patients had cessation of bleeding with no adverse events due to medication administration (13). Our institution has used intrapulmonary rFVIIa to treated 13 patients diagnosed with DAH, of which 6 survived the acute event (90). In these cohorts of patients who had cessation of DAH, there were no reported treatment related complications such as thromboembolic events or recurrence of DAH following treatment (10, 12, 38). While use of intrapulmonary rFVIIa to achieve hemostasis in DAH is encouraging, further prospective studies to evaluate its use and standardize dosing are needed.

### Extracorporeal Membrane Oxygenation (ECMO)

The presence of severe bleeding is often considered a contraindication for use of extracorporeal membrane oxygenation (ECMO) due to the need for systemic anticoagulation; however, there have been several reported cases of its successful use in DAH due to systemic vasculitides as a rescue therapy (91). Morris et al. reported a case of the successful use of VA ECMO for DAH following HCT in a patient with Hurler Syndrome. The patient was systemically heparinized and was also treated with methylprednisolone and aminocaproic acid without further hemorrhage. He was able to be decannulated and survived to hospital discharge (92). In our institution, we also had a case of pulmonary hemorrhage following HSCT that was successfully supported on ECMO (93). Given reports of the successful use of ECMO for DAH as well as our experience, we advocate for its consideration in certain patients and propose that pulmonary hemorrhage and HSCT are not absolute contraindications for ECMO support.

## Special Considerations

### Thrombotic Microangiopathy

While uncommon, DAH has been reported in association with transplant-associated thrombotic microangiopathy (TA-TMA) (94). TA-TMA is as subset of the thrombotic microangiopathies characterized by Coombs negative hemolysis, proteinuria, increase in serum LDH, renal and/or neurologic dysfunction, and systemic serositis (95). Treatment options for TA-TMA include plasma exchange, the anti-CD20 monoclonal antibody rituximab, and the complement inhibitor eculizumab (96). Etanercept, a TNF-α antagonist, has also been suggested as a therapy option for TA-TMA (97). TA-TMA carries a significant mortality up to 75% alone, or higher when present with DAH (94, 96). Thus, in cases of suspected TA-TMA associated with DAH, the careful addition of one of these therapies should be considered.

### Idiopathic Pneumonia Syndrome

Idiopathic pneumonia syndrome (IPS) is a non-infectious pulmonary complication of HCT which shares many clinical similarities with DAH, albeit without hemorrhage. It presents with findings of diffuse alveolar injury in the absence of lower respiratory tract infection, cardiac dysfunction, acute renal failure, or iatrogenic fluid overload (98). IPS is characterized by elevated levels of cytokines including IL-6, IL-8, Ang-2, and TNFR1, a surrogate marker for TNF-α (99). Etanercept, a TNF-α-binding protein, has been successfully used in combination with glucocorticoids (2 mg/kg/day MP equivalent) for the treatment of IPS in the pediatric population (99). In the DAH subset of IPS, higher doses of glucocorticoids may be considered.

### Infection

While DAH is classically considered a non-infectious complication of HSCT, the presence of an occult infection must be considered in cases of recurrent hemorrhage or lack of response to treatment. Agusti et al. reported a high rate (6/11) of concurrent infections on post-mortem exam in areas separate from hemorrhage, including herpes pneumonia, CMV pneumonitis, bacterial pneumonia, and aspergillosis, though BAL studies were negative for infectious etiologies in the 7 patients who had a bronchoscopy within 7 days of death (15). In a report by Heggen et al. post-mortem exam of one patient who died from DAH-associated progressive respiratory failure showed lung cultures positive for both yeast and bacteria despite BAL culture negative for infectious organism at time of DAH diagnosis (5). Thus, we recommend a low threshold for antimicrobial coverage of occult infections in cases of DAH even when BAL studies do not identify an infectious etiology. In cases of diagnostic dilemma despite less invasive means such as CT and bronchoscopy, we recommend early consideration of lung biopsy.

### Myelogenous Leukemia

Nanjappa et al. reported a case series of 5 patients with AML, two of whom had undergone allo-HCT, who developed DAH (100). While respiratory compromise in leukemia patients is recognized, it is likely that pulmonary hemorrhage is underestimated. On post-mortem exam of patients with leukemia, pulmonary hemorrhage was present in 74% of cases (101). Additionally, in patients with AML and acute respiratory failure of unknown etiology, pathologic findings of DAD/DAH were present in ~22% of cases (102). In a series of patients initially hospitalized for respiratory failure and subsequently diagnosed with M5 AML, BAL fluid was hemorrhagic in 7/15 patients undergoing bronchoscopy (103). In acute and chronic myelogenous leukemia, a pattern of DAD characterized by endothelial cell hyperplasia, interstitial edema, and the presence of interstitial lymphocytes, has been described following the initiation of chemotherapy. Lung injury occurs from lysis of leukemic cells and release of intracellular enzymes such as collagenases, cathepsin G, and lysozymes (104). In the setting of acute myelogenous leukemia, this mechanism may be responsible for the initial lung injury seen in the development of DAH.

## Conclusions

Diffuse alveolar hemorrhage continues to be a recognized complication of HCT which has historically carried significant morbidity and mortality. While survival has seemingly improved with newer therapeutic options, there remains ample opportunity for optimization in the holistic care of these patients. In our institution, early recognition, a standard approach to diagnosis and treatment, and protection against additional organ dysfunction/endothelial damage are cornerstone to our treatment approach. Further research is needed to better understand the pathogenesis of this complex disease process, evaluate current treatment options, and develop new therapies in order to continue to improve outcomes.

## Author Contributions

SG conceptualized the scope of manuscript and provided intellectual contributions. SG and KF performed the literature search, analysis, and wrote the original draft. SG, JM, and RM provided critical revisions and to the draft. All authors contributed to the manuscript revision, read, and approved the final submitted version.

## Conflict of Interest

The authors declare that the research was conducted in the absence of any commercial or financial relationships that could be construed as a potential conflict of interest.
